# Synthesis of Vertically-Aligned Zinc Oxide Nanowires and Their Application as a Photocatalyst

**DOI:** 10.3390/nano7010009

**Published:** 2017-01-11

**Authors:** Qiong Zhou, John Z. Wen, Pei Zhao, William A. Anderson

**Affiliations:** 1 Department of Mechanical & Mechatronics Engineering, University of Waterloo, Waterloo, ON N2L 3G1, Canada; tracey.zhou0903@gmail.com (Q.Z.); zhaopeiplane@gmail.com (P.Z.); 2 Department of Chemical Engineering, University of Waterloo, Waterloo, ON N2L 3G1, Canada; wanderson@uwaterloo.ca

**Keywords:** zinc oxide, nanowires, photocatalysis, methyl orange, hydrothermal synthesis

## Abstract

Vertically aligned zinc oxide (ZnO) nanowires were hydrothermally synthesized on a glass substrate with the assistance of a pre-coated ZnO seeding layer. The crystalline structure, morphology and transmission spectrum of the as-synthesized sample were characterized by X-ray diffraction (XRD), field-emission scanning electron microscopy (FE-SEM), and ultraviolet-visible (UV-Vis) spectrophotometry, respectively, indicating a wurzite ZnO material of approximately 100 nm wire diameter and absorbance at 425 nm and lower wavelengths. The photocatalytic activity of the sample was tested via the degradation of methyl orange in aqueous solution under UV-A irradiation. The synthesized nanowires showed a high photocatalytic activity, which increased up to 90% degradation in 2 h as pH was increased to 12. It was shown that the photocatalytic activity of the nanowires was proportional to the length to diameter ratio of the nanowires, which was in turn controlled by the growth time and grain size of the seed layer. Estimates suggest that diffusion into the regions between nanowires may be significantly hindered. Finally, the reusability of the prepared ZnO nanowire samples was also investigated, with results showing that the nanowires still showed 97% of its original photoactivity after ten cycles of use.

## 1. Introduction

Industrial effluents, as well as household wastewater, have been major sources of residual dye pollutants that enter the environment and are not readily biodegradable. Traditional ways of treatment, such as adsorption on activated carbon, chemical precipitation and separation, and coagulation are non-destructive methods and only transfer dyes from one phase to another, causing secondary pollution and requiring further treatment [1,2,3]. In recent years, extensive research has been done on semiconductor-based heterogeneous photocatalysis, which has been found to be very effective in degrading a wide range of organic pollutants into non-hazardous non-toxic byproducts under ultraviolet (UV) or visible light irradiation. The semiconductors such as titanium dioxide (TiO_2_), zinc oxide (ZnO) and zinc sulfide (ZnS) act as a photosensitizer to generate electron-hole pairs upon irradiation with a suitable wavelength. These can either recombine or react with other species separately to produce strong oxidizing agents like hydroxyl or super oxide radicals [4,5].

Although TiO_2_ has been widely used for many environmental applications due to a faster electron transfer rate [6], large-scale application of TiO_2_ in industrial wastewater treatment has generally not proven to be economical. ZnO, with a similar band gap energy and photodegradation mechanism as TiO_2_ appears to be a suitable alternative [7,8]. In fact, ZnO absorbs a larger fraction of the solar spectrum than TiO_2_ due to the existence of a larger number of inherent active surface defect sites, and hence exhibits a higher photoactivity under visible light [9]. It has also been reported that ZnO has a higher quantum efficiency and photocatalytic efficiency than TiO_2_ [10,11,12,13] due to its effectiveness in generation and separation of photon-induced electron-hole pairs [14,15]. ZnO is also abundant in nature, non-toxic, has a variety of production methods [16] and is easier to grow either in the form of powder [17] or on top of various substrates [18,19,20].

Up to now, much effort has been done to prepare the photocatalytic materials including ZnO and TiO_2_ in the form of fine powders, as a high surface area generally gives a high photocatalytic efficiency. However, these fine powders tend to aggregate in the solution leading to severely reduced effective surface area. Most importantly, the separation and recovery of the nanosized photocatalysts from the reaction suspension is highly difficult, which limits its practical use in industry [21]. One way to eliminate this issue is to immobilize the photocatalysts onto a fixed substrate, using various thin film deposition techniques [22,23].

Compared to the nanoparticles deposited on a flat surface, one-dimensional nanostructures, such as nanowires grown on a substrate, offer a larger surface-to-volume ratio, and hence a higher photocatalytic activity through enhanced adsorption of target organic molecules onto the catalyst surface [24]. Moreover, the recombination rate of photon-induced electron-hole pairs is much lower in 1D nanowires compared to the 0D nanoparticles [25], which further contributes to the enhanced photocatalytic efficiency of nanowires over nanoparticles. There are also other advantages in stabilized nanowire structures, such as a wide choice of substrate materials and geometries, which make them a good candidate as photocatalysts.

Therefore, it is of practical use to fabricate well-aligned ZnO nanowires on flat substrates, to avoid aggregation of the photocatalysts and to eliminate the difficulty of separation and recovery of the photocatalysts from the reaction mixture. To date, there are several methods available to synthesize ZnO nanostructures, such as vapor liquid solid (VLS) growth [26], chemical or physical vapor deposition (CVD/PVD) [27,28], pulsed laser deposition (PLD) [29]. However, most of these methods use severe conditions such as high temperature, high pressure, expensive materials and complex procedures [30]. Hydrothermal methods, on the other hand, have many advantages such as low cost, ease of handling, low energy consumption and scalability, and hence have recently received attention for the synthesis of 1D nanostructures [24,30]. In photocatalytic applications, hydrothermal methods are even more advantageous as hydrothermally grown ZnO nanowires have more inherent crystalline defects, primarily due to oxygen vacancies [31], which give the structures high photocatalytic activity under visible light even without doping with transition metals [32].

In this work, we prepared the well-aligned ZnO nanowires on glass substrates pre-coated with ZnO seeding layers, by adapting a hydrothermal method reported by Joo et al. [33]. The crystallinity, structure, and morphology of the ZnO nanowire arrays were characterized by X-ray diffraction (XRD) and field-emission scanning electron microscopy (FE-SEM). The photocatalytic efficiency of the prepared ZnO nanowires was investigated based on the photodegradation rates of methyl orange (MO) solution. The effects of pH and initial dye concentration of the reaction solution on the photocatalytic efficiency of the ZnO nanowire arrays were also explored.

## 2. Materials and Methods

### 2.1. Synthesis of ZnO Nanowires

In this work, all materials except deionized (DI) water and ethanol were used as received from Sigma-Aldrich (Oakville, ON, Canada) unless otherwise noted. DI water was used to make all the aqueous solutions. ZnO nanowires were synthesized using a hydrothermal method adapted from Joo et al. [33], in which two steps were involved. The first step was the fabrication of zinc oxide seed layers, which were deposited onto glass substrates with size of 25 by 51 mm. The glass slides were first cleaned using isopropanol, and then rinsed with DI water and dried. A sol-gel solution for ZnO seed layers were prepared using 0.7 M zinc acetate dihydrate [Zn(CH_3_COO)_2_·2H_2_O)] as the precursor, 0.7 M monoethanolamine as the stabilizer and ethanol as the solvent. The resultant solution was stirred for 10 min to yield a clear, homogeneous and transparent solution. The seeding layer was fabricated by spin coating of a sol-gel solution (0.7 M zinc acetate dehydrate and 0.7 M monoethanolamine in 100 mL ethanol) onto pre-cleaned glass substrates (2 × 1 × 1 mm) at 3000 rpm for 40 s. This was followed by curing on a 250 °C hotplate for 10 min to evaporate the solvent, remove the organic residuals, and improve ZnO particle adhesion onto the substrate.

The seeded substrate was then placed upside down in a 100 mL solution in a closed jar containing 10 mM zinc sulfate and 300 mM ammonium chloride, with pH adjusted to 11 by diluted sodium hydroxide solution. The jar was placed in a convection oven and held at 60 °C for 6 h. The hydrothermally treated samples were then rinsed with DI water several times to eliminate residual salts or amino complexes, and air dried.

### 2.2. Characterization

The crystalline structure of the synthesized ZnO nanowire arrays was analyzed by XRD using XPERT-PRO diffractometer system (PANalytical, St. Laurent, QC, Canada) with Cu Kα radiation (λ = 1.54056 Å) at 45 kV and 35 mA, by scanning from 20° to 90° (2θ) using a step size of 0.05° and 1.0 s per step. The morphology and size of the grown ZnO nanowires were characterized by FE-SEM (LEO 1550, Zeiss, Toronto, ON, Canada). Finally, the ultraviolet-visible (UV-Vis) transmission spectra of the synthesized ZnO nanowire arrays were recorded by the HP Hewlett Packard 8452A Diode Array Spectrophotometer (HP, Mississauga, ON, Canada) over the wavelength range of 200–800 nm.

### 2.3. Photocatalytic Activity Test

The photocatalytic performance of the samples was evaluated by measuring the photocatalyzed discoloration rate of the test dye MO in aqueous solution. A glass substrate with nanowires grown on top of it was placed in a Petri dish with a diameter of 60 mm and a depth of 15 mm, to which 10 mL of a 5 mg/L MO solution was added. The UV light source used was comprised of three 40 W Philips low-pressure UV-A fluorescent lamps with main emission wavelength at 365 nm and an incident light intensity of about 70 mW/cm^2^. The light intensity was measured without MO solution, while in the photocatalytic tests MO solution would absorb a part of the light flux. A blank test was carried out with the same photon flux and no ZnO photocatalysts, where degradation wasn’t observed. Therefore, even though the UV absorption of MO may initiate reaction and photosensitization, the degradation of MO is mainly caused by the presence of photocatalysis which will be shown in the following results. Prior to irradiation, the solution was stirred in dark for 10 min to ensure adequate adsorption of the dye onto the catalyst surface, as determined in preliminary tests where the change in concentration during this period was approximately 0.5 mg/L. The first sample was taken right after the dark adsorption period to determine the absorbance at 464 nm (*A*_0_), which was regarded as the initial concentration of MO (*C*_0_). Constant stirring of the solution was achieved by using a magnetic stirrer, and the petri dish was covered with a UV-A transparent cover to minimize evaporation of solvent. Samples were taken from the solution at regular time intervals and immediately analyzed to determine its instantaneous absorbance (*A*) at 464 nm, which is the maximum absorption wavelength of MO. After absorbance measurement, the samples were returned to the reaction solution and the irradiation continued. The degradation efficiency was calculated using Equation (1):

(1) $$\mathrm{Degradation}=\frac{C_{0}-C}{C_{0}}\times 100\%=\frac{A_{0}-A}{A_{0}}\times 100\%$$

where *C*_0_ and *C* are the initial and post-irradiation concentration of the dye, respectively; while *A*_0_ and *A* are the initial and post-irradiation absorbance of the MO solution at 464 nm, respectively.

## 3. Results and Discussion

### 3.1. Characterization of ZnO Nanowires

**Figure 1:**
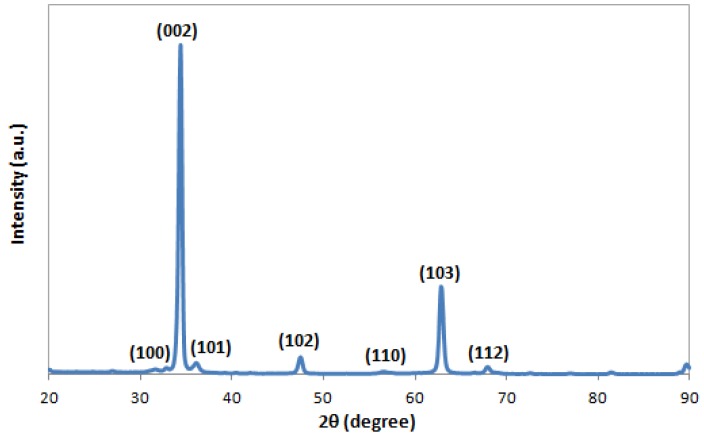
X-ray diffraction (XRD) patterns of the synthesized ZnO nanowire arrays.

Figure 1 depicts the XRD patterns of the as-prepared ZnO nanowire arrays grown on the glass substrate pre-seeded with ZnO nanoparticles using the hydrothermal method. It is observed that all the diffraction peaks are in good agreement with the standard ZnO hexagonal wurtzite crystalline structure on the JCPDS card, with measured lattice constants (*a* = *b* = 3.2498 Å, *c* = 5.2066 Å) being the same as the indexed ones [33]. A dominant diffraction peak for the (002) plane at 2θ = 34.43° indicates a high degree of anisotropic growth of ZnO nanowires along the *c*-axis vertical to the glass substrate surface. The peak is very strong and narrow, demonstrating a high degree of crystallinity of the prepared ZnO nanowires. Moreover, there are no other distinct peaks from impurities detected, indicating that the product is very pure. The results obtained from the XRD analysis are similar to the results reported by Joo et al. [33] and other groups [32,34,35,36].

**Figure 2:**
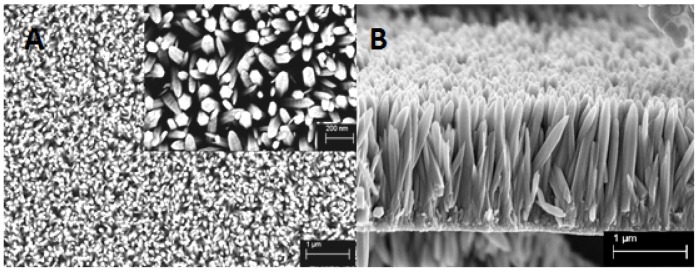
Scanning electron microscopy (SEM) images of the as-synthesized ZnO nanowire arrays: (**A**) top view; (**B**) cross-sectional view.

Figure 2 shows the SEM images of the prepared ZnO nanowire arrays, with both the top view and cross-sectional view. It can be easily seen that the nanowires are very well vertically aligned and closely packed onto the substrate, with an average diameter and length of approximately 100 nm and 1.5 μm, respectively. From the higher magnification image inserted in Figure 2A, it can also be observed that the synthesized nanowires demonstrate the hexagonal wurtzite structure, which confirms the XRD results in Figure 1, and are in good agreement with other publications about hydrothermal synthesis of ZnO nanowires [20,32,33,37].

**Figure 3:**
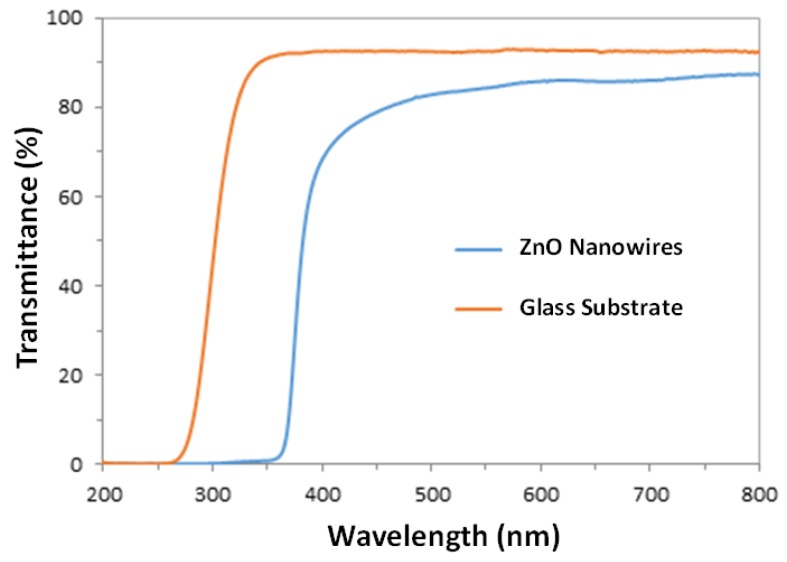
Ultraviolet-visible (UV-Vis) transmittance spectra of the prepared ZnO nanowire arrays and the standard glass substrate.

The UV-Visible transmittance spectra of the prepared ZnO nanowire arrays and the glass substrate are presented in Figure 3, which shows that the glass substrate is highly transparent (92% transmittance) in the visible region (400–700 nm) while the ZnO nanowires are capable of absorbing a small amount of visible light (86% transmittance). Both the ZnO nanowires and their glass substrate exhibit a sharp absorption band in the UV region (transition is 350–400 nm for ZnO nanowires, and 260–350 nm for glass slides). In this experiment, under the light source with main emission wavelength of 365 nm, 96% of the incoming light is absorbed by ZnO nanowire arrays for photocatalytic reactions, while the absorption by the glass substrate is negligible (8%).

### 3.2. Photocatalytic Activity of ZnO Nanowires

**Figure 4:**
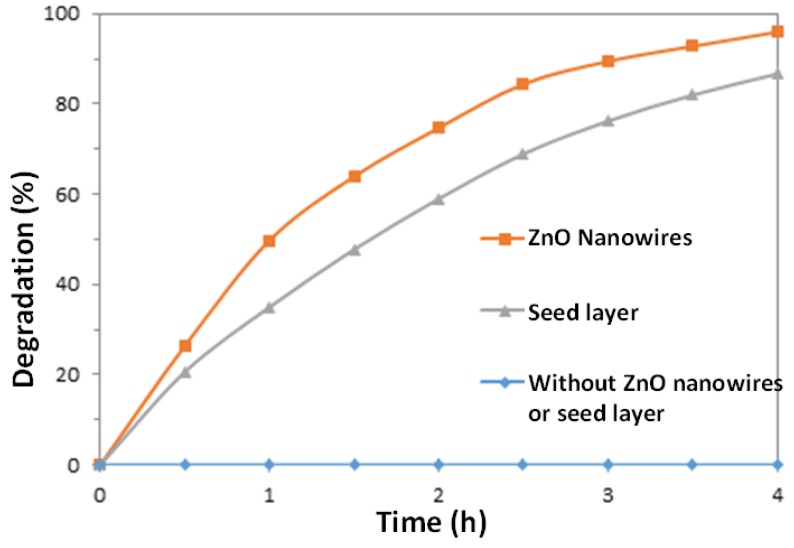
Photodegradation rates of methyl orange (MO) solutions in the presence of ZnO nanowires, ZnO seed layer, and without both.

Since some dyes can be degraded by direct UV irradiation without the assistance of catalysts [38], a blank experiment was carried out in the absence of the ZnO nanowire catalysts, while holding all other parameters the same. It can be seen from Figure 4 that in the presence of ZnO nanowires, 96% of dye was degraded after 4 h of irradiation; while in the presence of the ZnO seed layer, 86.7% of dye was degraded after 4 h. In contrast, there was no significant degradation of MO observed after 4 h for the same experiment performed in the absence of any ZnO material.

Reproducibility of the nanowire synthesis and photoreaction was tested using three replicate preparations. The MO degradation rates of the three samples were consistent throughout the experiment varying by only a maximum value of 3% over the 4 h reaction period, indicating that the synthesis method used in this study gives a very consistent result.

### 3.3. Effect of pH and Initial MO Concentration

**Figure 5:**
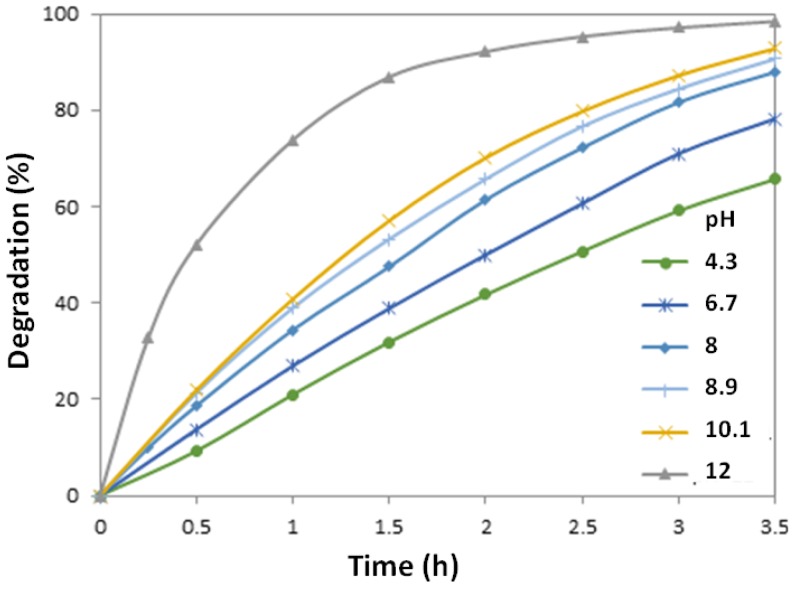
Photodegradation rates of MO solutions with different pH values catalyzed by ZnO nanowires prepared at the same conditions.

Due to the amphoteric property of many semiconductor oxides, it is very important to investigate the effect of pH in the dye solution on the reactions that take place on the semiconductor surfaces, as pH is a main factor that influences the surface charge profile of the photocatalysts [39]. Experiments were carried out in the pH range 4–12 in the aqueous dye solution. Figure 5 depicts the degradation rates of MO solutions with different pH values photocatalyzed with the prepared ZnO nanowire arrays. It is observed that the extent of photocatalysis increases with increasing pH, exhibiting a maximum rate of degradation at pH 12. Kansal et al. [40] observed similar results in their studies on pararosaniline chloride dye. A control experiment was also conducted using the same MO solution at pH 12 but without ZnO nanowire catalysts, to investigate the possibility of alkaline hydrolysis of MO. It was observed that no significant change occurred, indicating that the observed fast degradation rate of MO under pH 12 is only due to photocatalysis.

In an acidic environment, photodecomposition of ZnO takes place according to Equation (2):

(2) $$\mathrm{ZnO}+2H^{+}\to {\mathrm{Zn}}^{2+}+H_{2}O$$

The photocorrosion of ZnO is most rapid in a strong acidic environment (pH lower than 4) [41]. In an alkaline environment, photocorrosion of ZnO is less severe with increasing pH and no photocorrosion takes place at pH higher than 10 [41]. More importantly, in alkaline solution, large quantities of OH^−^ ions are present on the catalyst surface and in the reaction medium, which promotes the formation of hydroxyl radicals (·OH) [42,43], the species which have been widely accepted as a primary cause of organic dye degradation in photocatalytic reactions [3,11,12,44].

**Figure 6:**
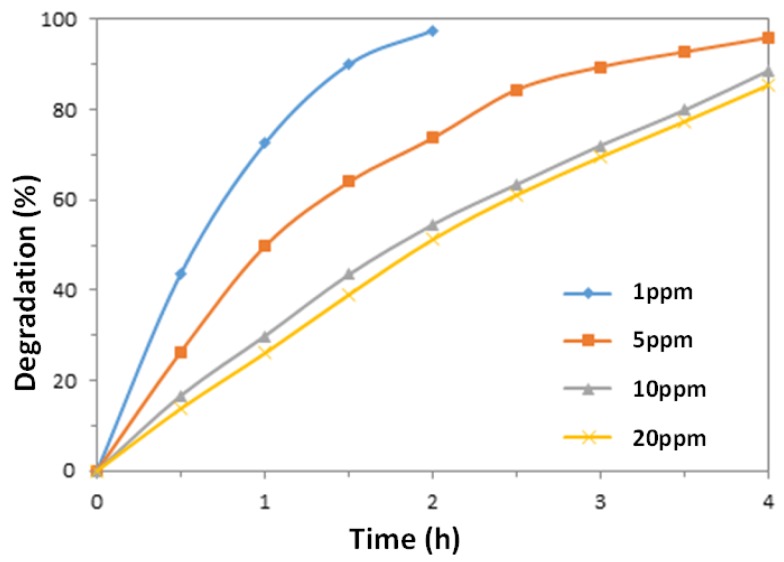
Photodegradation rates of MO solutions with different initial dye concentrations catalyzed by ZnO nanowires prepared at the same conditions.

Successful application of the photocatalytic degradation system requires investigation of the effect of initial dye concentration of the dye solutions on the photocatalytic efficiency, as industrial or household waste water comes in different concentrations. Figure 6 shows the photocatalytic degradation rates of MO solutions with different initial concentrations following the same treatment process. Since the reaction half-life (50% degradation) is not constant, it can be concluded that the system does not follow apparent first order kinetics.

The photocatalytic kinetics of many dyes has been studied with the Langmuir-Hinshelwood equation. With also considering the adsorption of the dye on the photocatalysts, this model is expressed as the following [45]:

*r* = *K*_reaction_*K*_adsoption_*C*/(1 + *K*_adsoption_*C*_0_) = *KC* (3)

**Figure 7:**
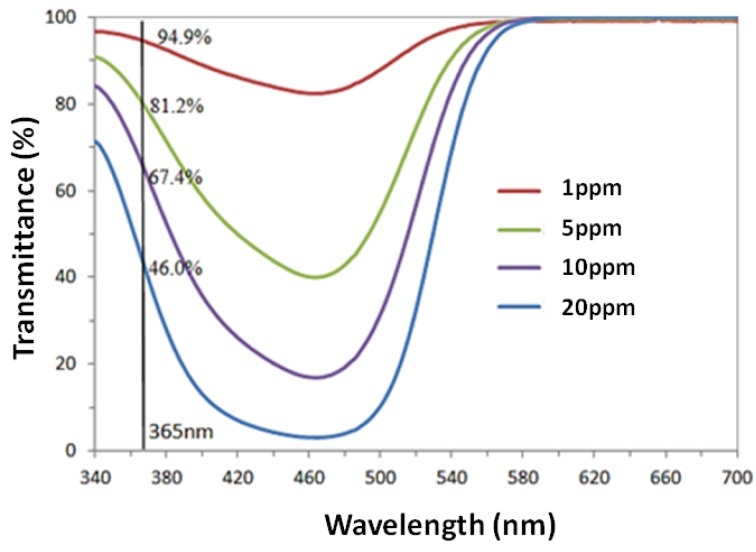
UV-Vis transmission spectra of MO solutions with different concentrations.

The kinetic constant *K* relates to reaction constant, adsorption constant and initial MO concentration, and thus is specific to each experimental system. From Equation (3), it is recognized that the kinetic constant increases with the decline of initial concentration, which agrees with the results in Figure 6, as a higher kinetic constant corresponds to a higher degradation at the same irradiation time. As the dye concentration increases, the consumption rate of highly active species including hydroxyl radicals (·OH) and superoxide anions ($O_{2}^{-}$) also increases [46]. However, the generation of the active species on the photocatalyst surface actually decreases with increasing dye concentrations, as a result of the reduction transmittance of the light at 365 nm shown in Figure 7. Moreover, slow diffusion of the generated intermediates from the catalyst surface can lead to the deactivation of the active sites on the photocatalyst surface, and may contribute to the reduction in the photodegradation efficiency with increasing dye concentrations [4].

### 3.4. Effect of Nanowire Growth Time

**Figure 8:**
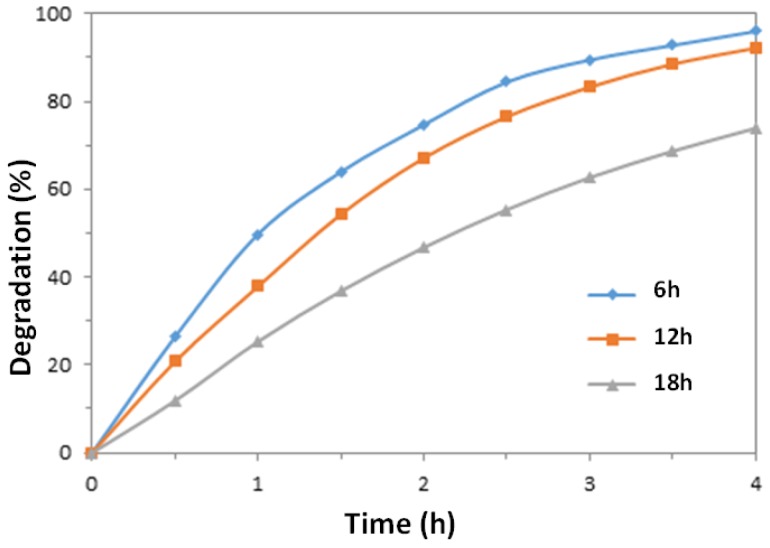
Photodegradation rates of MO solutions catalyzed by ZnO nanowires prepared for different growth durations (6, 12, and 18 h) in the precursor solutions at the same conditions.

Figure 8 shows the effect of growth time of the ZnO nanowires (6, 12, and 18 h) in the hydrothermal precursor solution on their photodegradation efficiency. It can be observed that with longer growth times, the obtained nanowire arrays exhibited decreasing photocatalytic reaction rates. As reported by Joo et al. [33], there is a rapid growth of ZnO nanowires in the first 4 h (initial stage), and then a low growth rate for up to 20 h (growth stage). The growth rate is slowed down after the initial stage due to a depletion of the precursors in the growth solution. With increasing growth duration time, it is reported that both length and diameter of the prepared nanowires are increased but the overall aspect ratio (*L*/*D*) is reduced [47]. In this experiment, the aspect ratios of the nanowires grown for different durations are found to be 15 for 6 h, 10 for 12 h and 8 for 18 h. Hence, with longer growth duration, the aspect ratio of the nanowires decreases, which gives a smaller photocatalytic surface-to-volume ratio.

On a simple surface area basis, the enhanced area can be estimated as follows. From the SEM micrographs, assume that an area of 1 µm × 1 µm contains approximately 49 vertical nanowires, with approximate dimensions of 50 nm diameter by 1500 nm height. Treating that the nanowire as cylinders, the total surface area of the 49 cylinders is approximately 23.5 µm^2^, versus the 1 µm^2^ of the base without nanowires. However, as indicated in Figure 4 there was not a 23.5 times enhancement of photocatalytic reaction rate for a nanowire surface over that of the ZnO seed layer alone. It is apparent that the effect of enhanced surface area by the nanowire geometry is complicated by mass and photon transfer issues.

To assess the magnitude of possible mass transfer effects, the influence of the nanowires on the MO reactant diffusivity along the nanowires was estimated by considering it as a pore diffusion problem and employing Ternan’s method [48] to find the ratio of the effective diffusivity in a liquid-filled pore (*D_eff_*) to the diffusivity of MO in bulk solution (*D_B_*), given as:

(4) $$\frac{D_{eff}}{D_{B}}=\frac{{\left(1-\lambda \right)}^{2}}{1+P\lambda }$$

where λ is the ratio of the molecular radius over the pore radius, and *P* is a parameter accounting for solution-wall interactions. The parameter *P* is given by the following:

(5) $$P=\left[2-\lambda +\beta /\lambda \left(2-2\lambda -\beta \right)\right]\frac{\Delta \mu_{w}}{\mu_{B}}$$

where $\beta =\frac{r_{w}}{r_{p}}$ and $\lambda =\frac{r_{m}}{r_{p}}$. To estimate *r_m_*, the radius of the MO, the molar volume (321 cm^3^/mol) was estimated using Le Bas additive volumes [49], which was then used to estimate the volume per molecule, and from that a radius of 0.5 nm, assuming a spherical molecule. The approximate distance between nanowires (200 nm) was chosen to represent a pore diameter (2 × *r_p_*), resulting in a value of λ of 5.0 × 10^−3^. For β, the distance from the pore wall in which solvent has enhanced viscosity, *r_w_*, was assumed to be one molecular diameter of water (i.e., 0.28 nm) [50], resulting in a value of β of 0.56. The ratio of $\frac{\Delta \mu_{w}}{\mu_{B}}$ is uncertain for this system, but a value of 95.6 was estimated for glucose diffusion in water-filled alumino-silicate pores [50], and this value was used here. With these assumptions, the value of *D_eff_*/*D_B_* was estimated to be approximately 0.4.

It seems likely, therefore, that the enhanced surface area due to the presence of nanowires is not entirely useable due to diffusion limitations, partially explaining the lack of proportional increase in photocatalytic rates. Longer growth periods for the nanowires, which resulted in lower length to diameter ratios, reduce the apparent “pore diameter” between nanowires, resulting in increased diffusion resistance and the decreasing photocatalytic activity shown in Figure 8. Additionally, for photocatalytic rate enhancement, penetration and distribution of UV light across all the surface area is also required [51]. For small scale nanowire features, where the dimensions are similar in magnitude to the UV wavelengths, geometric optics are not directly applicable [52] and solution of Maxwell’s equations is required, which was beyond the scope of this work. The work by Hu and Chen [52] for silicon nanowires suggests that these arrays have higher absorbance than thin films, which implies some distribution of the UV energy over the surface. Therefore, the combination of reduced mass transfer of molecules to the surfaces, and a spread of UV energy over a larger surface area, is a likely rationale for the lack of more significant rate enhancement when nanowire arrays are present.

### 3.5. Reusability of ZnO Nanowires

**Figure 9:**
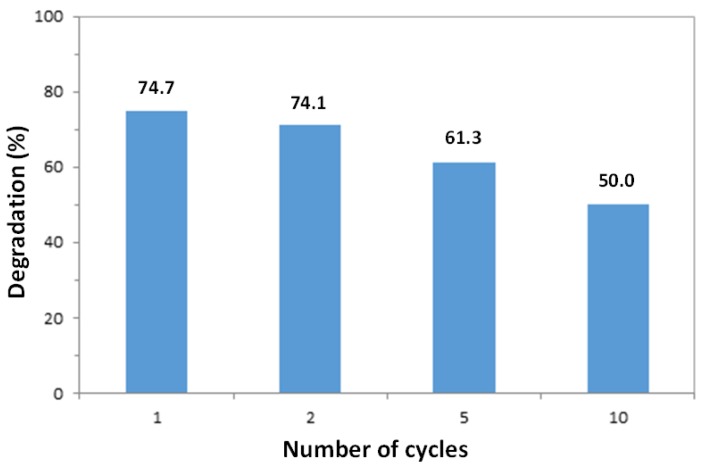
Percentage degradation values of MO solutions after 2 h of irradiation using the same ZnO nanowire sample after multiple cycles.

To evaluate the reusability of the synthesized ZnO nanowire arrays for photocatalytic applications, the glass substrate with aligned ZnO nanowires was collected after each photodegradation of a 10 mL MO solution (10 mg/L) for two hours, cleaned with DI water several times and blow-dried with air. The dried-catalyst sample was used again for degradation of a fresh dye solution following the same experimental conditions. The process was repeated up to ten times, and the percentage degradation data after two hours of irradiation was calculated based on the change in absorbance, as shown in Figure 9. It can be observed that the photocatalytic efficiency of the ZnO nanowire arrays only exhibited a small reduction in activity after each cycle (approximately 3%). The photocatalytic nanowires continued to show considerable photocatalytic activity even after ten cycles, which reveals the photostability of the synthesized photocatalyst and its potential for recycle and reuse. The chemical stability of ZnO nanowires (dissolution) was studied by other researchers. ZnO can be partially dissolved by DI water, ammonia, and NaOH solution, and smaller particles show a greater dissolution than the larger ones [53,54]. The presence of dye and UV markedly accelerates the corrosion rate of ZnO [55]. Thinner ZnO film shows a higher corrosion rate [55,56]. Etching pits on the surface of ZnO photocatalysts would commonly appear due to dissolution after photocatalytic reactions [55,57,58].

It is worth mentioning that the products of the degradation of MO were monitored in others’ studies [59,60]. The MO was decomposed to inorganic end products (carbon dioxide, SO_4_^2−^, NO_3_^−^, NH_4_^+^) through the formation of intermediates. Major intermediate species included hydroxylated derivatives, naphthoquinone, aromatic amines, and phenolic compounds.

## 4. Conclusions

Vertically-aligned ZnO nanowires were grown using a facile hydrothermal method, onto a glass substrate pre-coated with a thin ZnO seed layer deposited via spin-coating and annealing. The hydrothermally grown ZnO samples showed a hexagonal wurtzite structure with a high degree of anisotropy along the *c*-axis and a good crystallinity. The nanowires had an average diameter and length of approximately 100 nm and 1.5 μm, respectively, and were capable of absorbing 96% of a 365 nm light source. The ZnO nanowire samples exhibited a superior photocatalytic activity in terms of photodegradation of MO in aqueous solution, and the photoefficiency was found to be very consistent for samples prepared separately using the same method. The photodegradation rates of MO increased with higher pH of reaction solution, possibly due to a larger rate of formation of hydroxyl radicals. For different initial dye concentrations, the photodegradation rates were found to follow apparent Langmuir-Hinshelwood kinetics. Furthermore, with longer growth duration time, the synthesized ZnO nanowires showed a reduction in their photocatalytic efficiency due to a lower aspect ratio of the resulting nanowires, and the possible effects of mass transfer limitations. The ZnO nanowire samples were also reused for multiple cycles to test their reusability, and a high degree of photocatalytic activity was still present after ten cycles, which reveals the stability of the ZnO nanowire samples.

## Nomenclature

*C*	concentration (mg/L) of methyl orange
*k*	rate constant (mg/L·h)
*K*	kinetic constant (L/mg)
*D_eff_*	effective diffusivity in liquid-filled pore
*D_B_*	diffusivity in bulk solution
*P*	parameter in Equation 6 (dimensionless)
*r_m_*	radius of solute molecule (nm)
*r_p_*	radius of pore (nm)
*r_w_*	distance from pore wall where solvent has enhanced viscosity (nm)
Δμ*_w_*	enhanced viscosity of solvent near the pore wall (kg/m·s)
μ*_B_*	viscosity of bulk solvent (kg/m·s)
β	*r_w_*/*r_p_* (dimensionless)
λ	*r_m_*/*r_p_* (dimensionless)
